# Sequential targeted therapy in synchronous dual-primary lung adenocarcinomas with EGFR and RET alterations: a 5-year follow-up case report

**DOI:** 10.3389/fonc.2026.1769932

**Published:** 2026-02-25

**Authors:** Yong-liang Niu, Ying Yang, Xiao-bao Teng, Ming-feng Han, Di-ming Wang

**Affiliations:** 1 Department of Respiratory and Critical Care Medicine, No.2 People’s Hospital of Fuyang City, Fuyang Infectious Disease Clinical College of Anhui Medical University, Fuyang, China; 2 Shanghai Lung Cancer Center, Shanghai Chest Hospital, Shanghai Jiao Tong University, Shanghai, China; 3 Department of Oncology, Anhui Chest Hospital, Hefei, China

**Keywords:** anlotinib, dual-primary lung adenocarcinoma, EGFR mutation, intratumoral heterogeneity, pralsetinib, RET fusion

## Abstract

With the increasing detection of multiple primary pulmonary nodules, accurately distinguishing between multiple primary lung cancers and intrapulmonary metastasis is crucial for diagnosis and treatment. We herein report a case of a 71-year-old female with bilateral multiple primary lung adenocarcinomas, in which separate lesions harbored an EGFR 19del mutation and a RET fusion gene, demonstrating intratumoral genetic heterogeneity. The patient was successively treated with an EGFR-TKI, chemotherapy, and the RET inhibitor pralsetinib, the latter of which maintained a response for over three years. Following the development of resistance, combination therapy with pralsetinib and anlotinib successfully achieved a partial response again. This case underscores the importance of comprehensive molecular testing across multiple lesions to guide precision therapy and provides clinical insights into RET fusion-positive lung cancer treatment and post-resistance combination strategies.

## Introduction

Globally, lung cancer continues to be the most frequently diagnosed malignancy and the leading cause of cancer-related deaths (1). The increasing utilization of low-dose computed tomography (CT) for screening high-risk populations, coupled with broader access to high-resolution CT and positron emission tomography–CT (PET-CT), has resulted in a rising clinical detection of multiple pulmonary tumor nodules (MPTN) (2). Epidermal growth factor receptor (EGFR) mutations represent the most prevalent oncogenic driver in non-small cell lung cancer (NSCLC) among East Asian populations, particularly in patients with adenocarcinoma histology, never- or light-smoking status, and female sex. In China, EGFR mutations are detected in approximately 50%–60% of lung adenocarcinoma cases—substantially higher than in Western cohorts—and predominantly consist of exon 19 deletions and the L858R point mutation in exon 21, which confer sensitivity to EGFR tyrosine kinase inhibitors (TKIs) (3).RET fusion is a recognized oncogenic driver, identified in approximately 1% to 2% of patients with NSCLC (4). Notably, in addition to *de novo* RET fusions, acquired RET fusion has been reported as a resistance mechanism to third-generation EGFR-TKIs, accounting for about 2% of such resistance cases (5). Current therapeutic strategies primarily rely on RET inhibitors. However, multi-primary lung adenocarcinomas harboring concurrent EGFR mutations and RET fusions have been rarely reported in the literature, and their optimal treatment strategy remains unclear. Here, we report a case of dual-primary lung adenocarcinoma with concomitant EGFR mutation and RET fusion. The patient was managed with a sequence of therapies including EGFR-TKIs, chemotherapy, and RET inhibitors, and has achieved an overall survival exceeding five years to date.

## Case report

**Figure 1:**
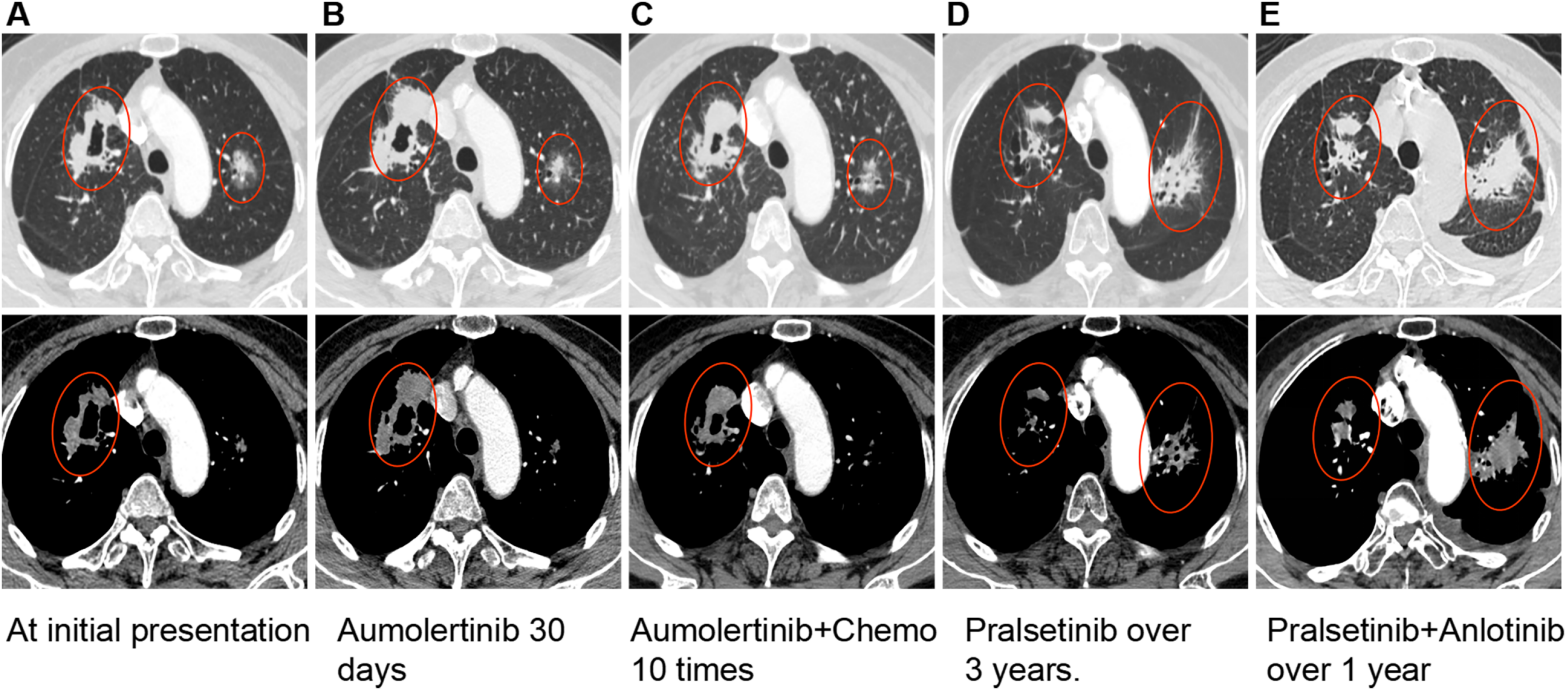
Serial chest CT scans showing the evolution of bilateral lung lesions. **(A)** At initial diagnosis; **(B)** One month after initiation of aumolertinib; **(C)** At approximately 10 months after diagnosis, demonstrating disease progression; **(D)** At approximately 52 months after diagnosis (nearly 3 years after starting pralsetinib), showing response in the right lung lesion but progression in the left; **(E)** At approximately 64 months after diagnosis (12 months after initiating pralsetinib plus anlotinib), revealing further enlargement of the left lung lesion with stable disease in the right.

**Figure 2:**
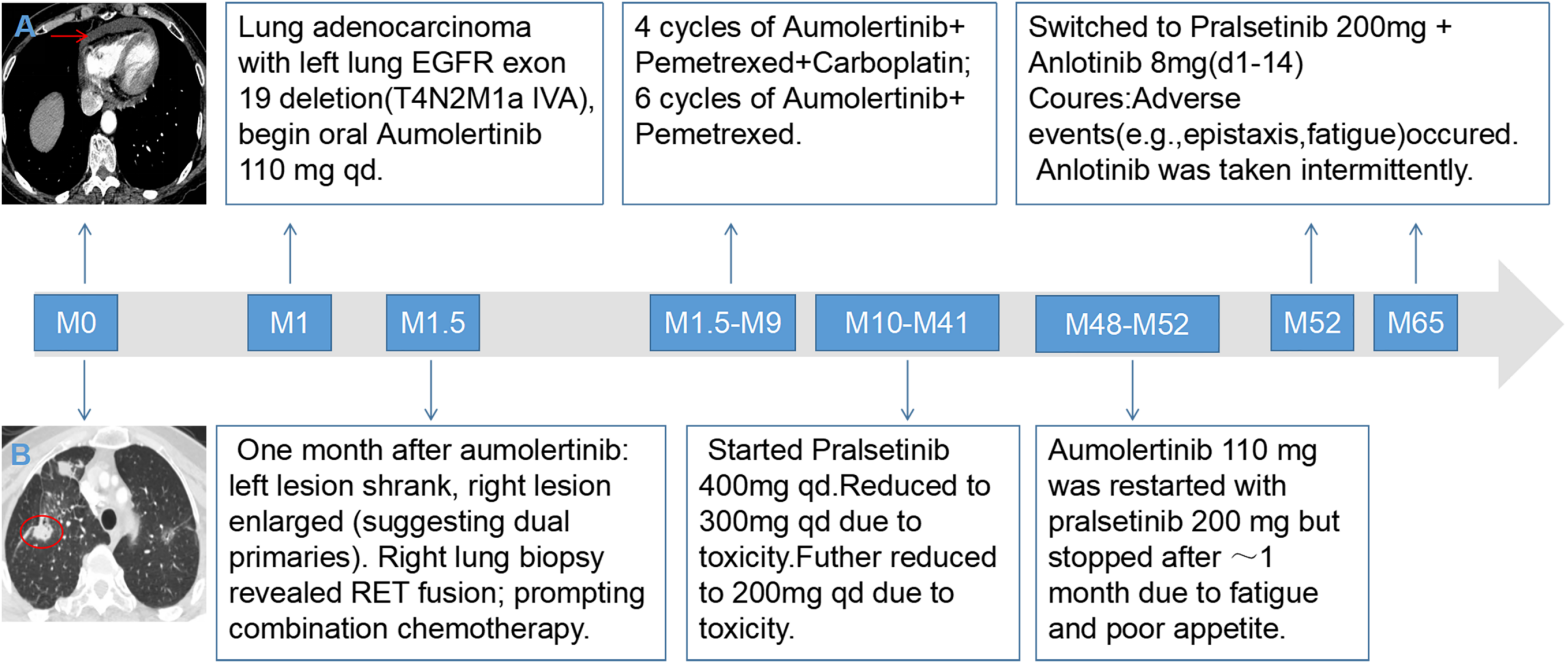
Summary of Treatment Sequence. **(A)** Mediastinal window CT scan at initial diagnosis showing pericardial effusion (indicated by red arrow). **(B)** Lung window CT scan at initial diagnosis showing intrapulmonary disseminated nodules (indicated by red arrow).• Diagnosis: Synchronous dual-primary lung adenocarcinoma (Left: EGFR 19del; Right: RET fusion).• 1st Line (EGFR-targeted): Aumolertinib → right lesion progressed, leading to diagnosis revision.• 1st Line (Intensified): Added platinum-pemetrexed chemotherapy targeting both lesions.• 2nd Line (RET-targeted): Switched to pralsetinib upon progression. Dose reduced (400mg→200mg) due to toxicity, maintaining efficacy for ~3 years.• 3rd Line (Combination): Upon new progression, anlotinib added to pralsetinib.

A 71-year-old Asian female non-smoker presented with a non-productive cough and occasional bloody sputum of unknown cause, which did not respond to anti-infective and antitussive treatments. Initial evaluation at an external hospital raised concern for tuberculosis due to her husband’s history of active pulmonary tuberculosis; however, sputum acid-fast bacillus smear and T-SPOT tests were negative. At the time of referral, a chest non-contrast CT revealed a right lung mass. A contrast-enhanced chest CT performed at diagnosis (**Figure 1A**) demonstrated an irregular cavitary lesion in the apical segment of the right upper lobe and a mixed ground-glass opacity nodule in the apicoposterior segment of the left upper lobe. Additionally, multiple miliary nodules were observed bilaterally, accompanied by pericardial effusion (**Figure 2A**) and suspected pleural metastatic nodules (**Figure 2B**), raising suspicion for a malignant neoplasm. PET/CT confirmed FDG-avid lesions: a lobulated thick-walled cavity in the right upper lobe (approximately 5.3 × 2.9 cm) and a mixed ground-glass opacity in the left upper lobe (approximately 2.6 × 1.8 cm), along with multiple metastatic lymph nodes in the mediastinum and hila.

Navigational bronchoscopy (ENB-EBUS-GS-TBLB) was performed. Biopsy from the left upper lobe apicoposterior segment mass confirmed adenocarcinoma with acinar and lepidic patterns. Immunohistochemistry (IHC): TTF-1(+), NapsinA(+), CK(+), P40(−), CD56(−). Molecular testing on this specimen revealed an EGFR exon 19 deletion, with no alterations in K-ras, B-raf, ALK, or ROS1. PD-L1 expression was 7%. Biopsy from the right upper lobe anterior segment showed bronchial mucosa with scant interstitial adenocarcinoma components and scattered nearby adenocarcinoma cells, with identical IHC profile (TTF-1+, NapsinA+, CK+, P40−, CD56−). According to the 7th edition of the AJCC TNM classification, the patient was initially diagnosed with lung adenocarcinoma staged as T4N2M1a (Stage IV) involving both lungs; an EGFR exon 19 deletion was detected in the left-sided lesion.

The patient initiated treatment with aumolertinib (110 mg once daily). A follow-up chest CT performed one month after starting therapy (**Figure 1B**) showed stable disease with a 10% reduction in the left lung lesion, whereas the right lung lesion had increased in size by approximately 15%, suggesting tumor heterogeneity. Given this discordant response, additional molecular testing was performed on the archival biopsy specimen from the right upper lobe lesion obtained at initial diagnostic bronchoscopy. This analysis identified a RET fusion (KIF5B-RET and RXFP1-RET) with wild-type EGFR, leading to a revised diagnosis of synchronous dual-primary lung adenocarcinomas: left lung harboring EGFR exon 19 deletion and right lung driven by RET fusion.

Approximately three months after diagnosis, the patient began combination therapy with aumolertinib and four cycles of pemetrexed/carboplatin chemotherapy, followed by maintenance therapy with aumolertinib plus pemetrexed. During this period, the left lung lesion remained stable, while the right lung lesion continued to progress. Overall disease progression was documented at approximately 10 months after initial diagnosis (**Figure 1C**).

When switching to pralsetinib treatment, we considered that the right lung RET-fusion lesion was an independent primary site and had shown no response to aumolertinib, while the left lung EGFR-mutant lesion remained stable at that time. Therefore, we decided to temporarily suspend the EGFR-TKI and prioritize potent inhibition of the progressing RET-fusion lesion. In addition, combining two targeted agents could increase the potential risk of overlapping toxicities.

Treatment was then switched to pralsetinib (400 mg once daily). Due to toxicities-including grade II myelosuppression, limb numbness, and thrombocytopenia-the dose was gradually reduced and maintained at 200 mg once daily. The right lung lesion achieved sustained remission for nearly three years on pralsetinib monotherapy. However, a chest CT performed nearly three years after initiating pralsetinib (**Figure 1D**) showed a partial response in the right lung lesion but significant enlargement of the left lung lesion, indicating progression of the EGFR-driven component. Between months 48 and 52 after initial diagnosis, the patient was re-challenged with aumolertinib in combination with pralsetinib; however, treatment was discontinued due to severe fatigue and poor appetite that were not tolerable.

Accordingly, approximately 52 months after initial diagnosis, the regimen was adjusted to pralsetinib (200 mg once daily) combined with anlotinib (8 mg on days 1–14 of a 21-day cycle). One year after initiating pralsetinib plus anlotinib (approximately 64 months after initial diagnosis), follow-up imaging (**Figure 1E**) revealed further enlargement of the left lung lesion, confirming disease progression in the EGFR-mutant component, while the right-sided RET-rearranged lesion remained stable. A summary of the treatment timeline is provided in **Figure 2**. All timepoints are expressed relative to the date of initial diagnosis (designated as month 0).

## Discussion

This case illustrates a rare and clinically instructive instance of synchronous dual-primary lung adenocarcinoma, harboring distinct driver alterations—EGFR exon 19 deletion in the left upper lobe lesion and a RET fusion in the right upper lobe lesion. Comprehensive molecular profiling of each lesion was pivotal in establishing the diagnosis and guiding a sequential, precision-based therapeutic approach, ultimately contributing to an overall survival exceeding five years. This experience underscores the critical importance of performing independent molecular characterization in patients with multifocal lung tumors to inform accurate staging and treatment decisions.

The increasing detection of multiple pulmonary nodules presents a diagnostic challenge in distinguishing multiple primary lung cancers (MPLCs) from intrapulmonary metastases (IPMs). As emphasized in recent International Association for the Study of Lung Cancer (IASLC) guidelines, large-scale next-generation sequencing (NGS) has become an indispensable tool in the diagnostic workup of such cases, providing key molecular evidence for differentiation (6). In our patient, the discordant driver mutational status (EGFR vs. RET) between the bilateral lesions strongly supported the diagnosis of synchronous MPLCs rather than metastatic disease. This molecular distinction directly influenced the therapeutic strategy, moving beyond a uniform treatment for a single metastatic cancer toward individualized targeting of each distinct primary tumor. Notably, a recently published case from the LIBRETTO-321 trial reported a treatment-naïve Chinese patient with advanced NSCLC harboring concurrent EGFR exon 19 deletion and KIF5B-RET fusion within the same tumor (7). Despite the presence of an EGFR mutation, the patient achieved a partial response lasting 14.7 months with first-line selpercatinib monotherapy, suggesting that the RET fusion may have been the dominant oncogenic driver. In contrast, our patient presented with synchronous dual-primary lung adenocarcinomas, with spatially distinct EGFR-mutant and RET-rearranged lesions. This anatomical and molecular separation necessitated a sequential therapeutic approach rather than single-agent targeted therapy. The differential response to initial EGFR-TKI—stabilization of the left lesion versus progression of the right—further supports the biological independence of these two primaries. Together, these cases highlight the critical importance of lesion-specific molecular profiling in multifocal lung cancer, as therapeutic strategies must be tailored to the underlying genomic architecture of each individual tumor.

The management of advanced NSCLC has been revolutionized by targeted therapies against actionable driver mutations. For EGFR-mutant NSCLC, third-generation TKIs such as Aumolertinib and Furmonertinib represent a standard first-line option (8, 9). Concurrently, RET fusions, found in 1–2% of NSCLC cases, define a distinct molecular subtype for which selective RET inhibitors like pralsetinib have demonstrated marked efficacy (4, 10). While RET fusions are typically mutually exclusive with other drivers like EGFR (11), they can emerge as a mechanism of acquired resistance to EGFR-TKIs (5). For such acquired RET fusions, combination therapy with an EGFR-TKI and a RET inhibitor has been established as an effective strategy, as evidenced by prior case reports (12). Notably, this patient received a short course of combination therapy with aumolertinib and a RET inhibitor at week 48 of treatment but developed severe anorexia and fatigue, leading to discontinuation. This highlights that the toxicity profile of dual-targeted inhibition warrants careful attention. However, the co-occurrence of *de novo* EGFR mutations and RET fusions in synchronous primary tumors is exceptionally rare, with no established treatment paradigm.

Our case provides novel insights into this clinical scenario. The RET fusion-positive lesion did not respond to initial EGFR-TKI therapy, highlighting its biological independence. Subsequent treatment with the selective RET inhibitor pralsetinib induced a partial response that was maintained for over three years, marking, to our knowledge, the first report of prolonged efficacy of pralsetinib in the context of a synchronous dual-primary adenocarcinoma. This finding expands the potential clinical application of pralsetinib beyond acquired resistance or solitary RET-driven tumors. In this case, the patient started pralsetinib at 400 mg once daily on June 19, 2021. Within one month, she experienced grade II myelosuppression—manifested as reductions in white blood cells, neutrophils, and platelets—along with limb numbness and other adverse events. Accordingly, the dose was lowered to 300 mg once daily after the first month. One year later, due to recurrent grade II myelosuppression, the dose was further reduced to 200 mg once daily. She was maintained on this dose for over two years, during which the right lung lesion remained in remission. This dose-adjustment course is consistent with the known safety profile and management principles of pralsetinib. Published data indicate that the most common grade ≥3 adverse events associated with pralsetinib include neutropenia (33%), hypertension (12%), and elevated liver enzymes, most of which can be effectively managed by dose interruption or reduction (13). Although our patient required multiple dose reductions owing to myelosuppression, long-term disease control was sustained at 200 mg, suggesting that pralsetinib can retain clinical efficacy even below the standard recommended dose. This finding aligns with real-world evidence indicating that dose adjustments do not compromise long-term outcomes (13). Moreover, another study on RET inhibitors reported that nearly half of the patients required dose reduction due to adverse events, yet treatment benefit was maintained thereafter (14). The present case further highlights the importance of individualized dose adjustment in enabling prolonged therapy, particularly in older or less tolerant patients. Furthermore, upon progression in the EGFR-mutant lesion, a combination of pralsetinib and the antiangiogenic agent anlotinib successfully re-achieved a partial response, suggesting a viable post-resistance strategy that warrants further investigation.

In this case, following progression of the left lung lesion harboring an EGFR mutation during pralsetinib monotherapy, we chose to combine anlotinib rather than continue long-term combination with an EGFR-TKI or chemotherapy, based on the following considerations. First, the patient had previously experienced severe anorexia and fatigue that were poorly tolerated during a brief trial of aumolertinib at 48 weeks post-diagnosis. Second, anlotinib, as a multi-target tyrosine kinase inhibitor, acts not only on VEGFR but also on pathways such as FGFR and PDGFR, potentially overcoming certain resistance mechanisms through anti-angiogenesis and modulation of the tumor microenvironment (15). Oral administration of anlotinib also offers greater convenience in clinical management, and the agent has demonstrated favorable tolerability as a third-line therapy in Chinese patients with advanced NSCLC (15). Nevertheless, the efficacy of this combination in dual-primary lung cancer with coexisting RET fusion and EGFR mutation remains anecdotal, and its underlying mechanisms require further exploration.

This single case report provides novel insights into the sequential targeting of heterogeneous drivers (EGFR and RET) in synchronous dual-primary lung adenocarcinomas; however, several limitations warrant acknowledgment. First, the observation from a single patient does not allow generalizable conclusions regarding the efficacy of low-dose pralsetinib (200 mg) in RET-fusion-positive lung cancer. The long-term effectiveness and dose–response relationship require validation in prospective cohorts. Second, the combination of pralsetinib and anlotinib was chosen based on individualized clinical judgment, and its use lacks prospective clinical evidence. The underlying mechanistic rationale and optimal patient population for this combination remain unclear. Finally, the treatment decisions in this case were highly personalized, influenced by factors including patient tolerance and drug accessibility; therefore, this approach cannot yet be regarded as a standard therapeutic paradigm. Future multi-center clinical studies or real-world data analyses are needed to further validate the safety and efficacy of such combination strategies.

In conclusion, this case highlights that synchronous dual-primary lung cancers with divergent driver mutations necessitate individualized treatment plans guided by comprehensive molecular profiling. Pralsetinib can deliver durable clinical benefit in RET fusion-positive primary lung cancer, even in the presence of a concurrent, independent EGFR-mutant primary tumor. Looking forward, integrating more refined analyses, such as clonality assessment to elucidate the evolutionary relationship between multifocal lesions, may further optimize therapeutic sequencing and combination strategies for patients with complex molecular landscapes (16).

## Data Availability

The raw data supporting the conclusions of this article will be made available by the authors, without undue reservation.
